# Supplementary material for: Impaired slow-wave sleep accounts for brain aging-related increases in anxiety
Source: Commun Psychol. 2026 Feb 4;4:34. doi: 10.1038/s44271-026-00401-2 (PMC12917002; doi:10.1038/s44271-026-00401-2)
Supplement: Supplementary file 2 — Supplementary Information [file 44271_2026_401_MOESM2_ESM.pdf]

Supplementary Information

Impaired slow-wave sleep accounts for brain aging-related increases in anxiety

Eti Ben Simon, Vyoma D. Shah, Olivia Murillo, Zsofia Zavecz and Matthew Walker

*Correspondence: Eti Ben Simon etibens@berkeley.edu

Table S1. Brain volume of key functional networks and next-day anxiety/SWA associations

Functional Network	Volume (mean ± SD)	Overnight Anxiety Association (r)	SWA Association (log count, r)
Visual	4.17 ± 0.41	-0.18 (*P* = 0.2)	0.19 (*P* = 0.16)
Somatomotor	3.99 ± 0.34	-0.07 (*P* = 0.6)	0.18 (*P* = 0.17)
Attention (dorsal)	2.72 ± 0.22	-0.13 (*P* = 0.36)	0.28 (**P* = 0.04)
Attention (ventral)	2.87 ± 0.23	-0.22 (*P* = 0.11)	0.25 (*P* = 0.065)
Limbic	2.95 ± 0.27	-0.09 (*P* = 0.5)	0.17 (*P* = 0.21)
Frontoparietal	3.75 ± 0.3	-0.17 (*P* = 0.2)	0.26 (*P* = 0.056)
Default Mode	6.42 ± 0.5	-0.2 (*P* = 0.15)	0.33 (**P* = 0.01)

SWA = Slow Wave Activity, Network volumes normalized for Total Intracranial Volume. * $P < 0.05$

Table S2. Age-based analysis of main study variables

Covariate (mean ± SD)	69 and younger	70-77	over 78	age-based ANOVA
Trait Anxiety	28.17 ± 2.64	28.31 ± 5.58	29.19 ± 6.57	$F(2, 55) = 0.167$, $P = 0.85$
Volume in anxiety-sensitive regions	1.83 ± 0.08	1.69 ± 0.1	1.61 ± 0.15	$F(2, 52) = 7.129$, $*P = 0.002$
SWA (log count)	2.58 ± 0.15	2.37 ± 0.23	2.34 ± 0.22	$F(2, 58) = 3.66$, $*P = 0.032$

SWA = Slow Wave Activity, ROI volumes normalized for Total Intracranial Volume. * P < 0.05

Fig-S1. Topographical distribution of NREM slow-wave parameters.

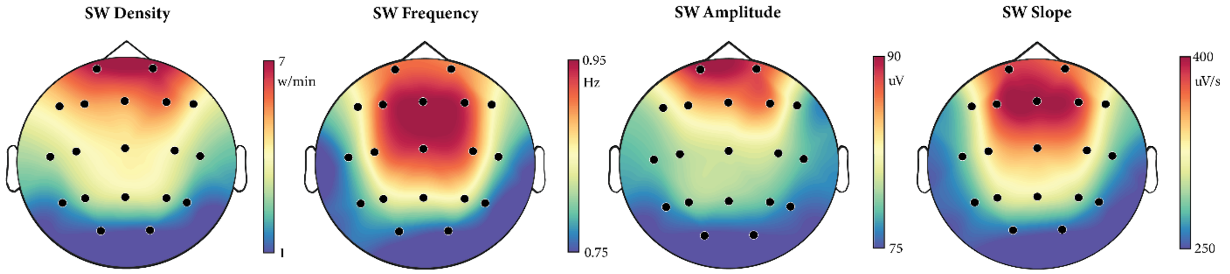

NREM slow-wave parameters at first visit averaged across participants (N=61, median average). Here, only channels with at least 20 detections across NREM sleep were included for each participant.

Fig-S2. NREM Spindles and Anxiety.

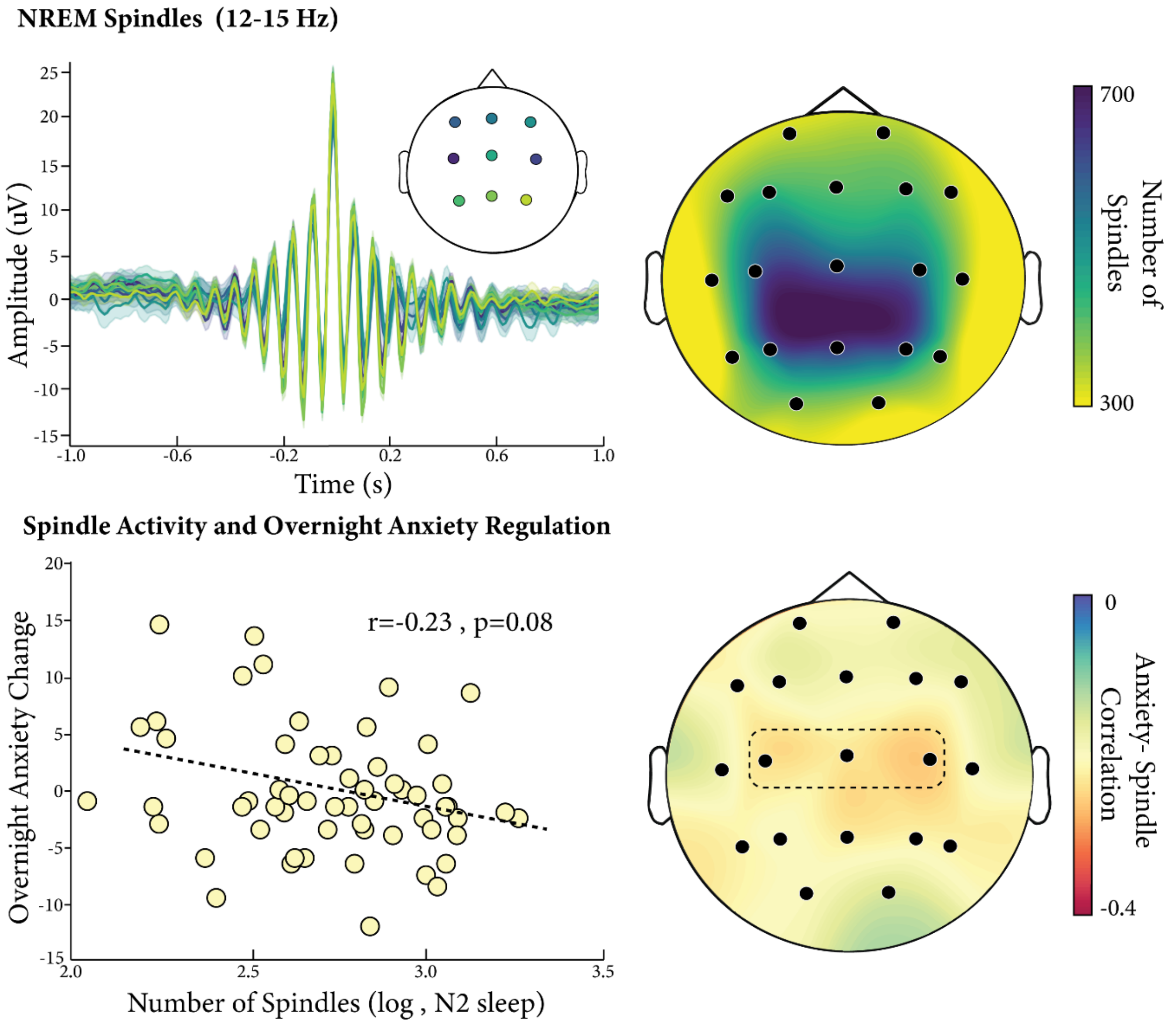

(**A**) Detected spindles from NREM sleep (left panel, single subject example) and average spindle counts across participants (N=61, right panel).

(**B**) Increased number of spindles during NREM2 sleep was not significantly associated with lower next-day anxiety (N=58, R=-0.23, *P* = 0.08, left panel) in either a predefined central derivative (right panel, marked in a dotted line) or across the scalp (right panel).

Fig-S3. Longitudinal changes in NREM SWA and anxiety.

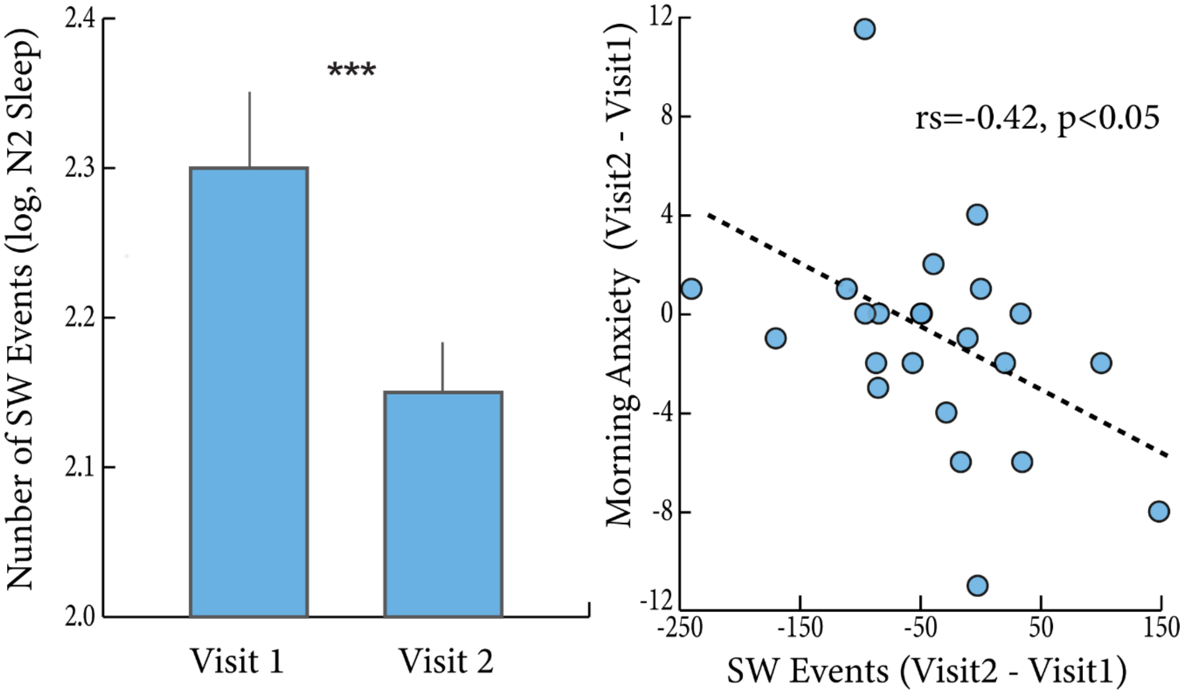

The number of NREM slow waves was significantly reduced from the first to the second visit (left panel, central derivative), a decrease that was associated with elevated anxiety levels at follow-up (*Rs* = -0.42, *P* < 0.05, N=23). *** *P* < 0.005
